# Modern Medico-Psychology and Psychiatry

**Published:** 1895-06-29

**Authors:** T. S. Clouston

**Affiliations:** Lecturer on Mental Diseases, Edinburgh University, and Physician Superintendent Royal Asylum, Morningside


### Modern Medico-Psychology and Psychiatry.

By T. S. Clouston, M.D., Lecturer on Mental Diseases, Edinburgh University, and Physician Superintendent Royal Asylum, Morningside.

### PARTIAL ARRESTMENTS OF BRAIN DEVELOPMENT.

The different parts of the brain, the great regulator of all the functions of body and mind, are developed in a certain order and sequence in the normal man and woman. Each part and each function has a due relation to every other part and every other function in development. This relational view of the functions of the brain is essential to a true understanding of development. Some organs and tissues of the body are brought to perfection early in life; others are very late in attaining maturity. The red corpuscles of the blood are perfected long before birth. The blood-vessels and the heart are early ready for their work. The lungs are ready for use the moment independent existence begins, but, though able to do their work, they remain unresistive to certain hostile agencies, such as the tubercle bacillus, till after full maturity, especially if there is a bad heredity. A child's skin is subject to the attacks of the ringworm spore, because it has not attained full development; that of the adult is not so, because it is mature. Probably this results from a want of trophic nerve force in the nerves that supply it and control its nutrition. We do not know as yet fully whether the special liability of the young to the whole class of zymotic diseases arises from imperfect brain development or not, but it is a reasonable hypothesis that, as no tissue and organ development can go on normally without full and proper innervation from the brain, and as the brain is probably not in a state of full maturity till the age of twenty-five, or so, the liability of many organs to special diseases during childhood and adolescence is due to imperfect brain development. We know as a matter of fact that any arrest in the maturing of the brain is often followed by special liability to diseases of many of the bodily organs. It is a well-known fact that the lymphatic glands and the lungs of idiots and imbeciles, where there has been a general arrest of brain growth or function, are especially liable to be attacked by the tubercle bacillus, so that two-thirds of all idiots die of tubercular disease. It is also a well-known fact that if we have a child with partial paralysis of one side of the body from arrested development or disease of the motor centres of the opposite side of the brain, the skin, the muscles, the blood-vessels, and the bones of that side will show deficient nutrition which will be lower in temperature, will not heal so well as the tissues on the unaffected side, and will take on diseased action more readily.

It is in the mental areas of the brain that arrests of development are best studied. Out of such arrests may arise very many serious social and legal consequences. They have also a high medico-psychological interest. When from partially arrested nervous energy supplied to the body, we have conditions of dwarfishness, ugliness, asymmetry of face and head, hunchbackness, and other deformities, we commonly have marked mental and moral peculiarities. There has in all ages been a prejudice against the morals and trustworthiness of such persons, and we know that some of them have been monsters of cruelty; of course, there are exceptions, but mental weakness or mental perversions undoubtedly prevail among them. A very curious result happens when we have a well-developed body, a handsome and expressive face and an imbecile condition of mind. This conjunction does occur in rare cases, and it can only do so through an unrelational development of different parts of the brain that ought to have acted in harmony with each other. The mental areas of the brain have in such cases suffered from arrested development while the centres regulating the nutrition of the bodily organs for the expression of mind, the face and eyes, have gone on to perfection. It is as when a watchmaker turns out a beautifully finished face and hands to his watch but puts in poor and untrustworthy works inside. The same kind of result is seen from the same cause when we have good intellectual power but no moral sense, no feeling of responsibility, and none of the higher social instincts. I have met with a great many such instances. They are the cause of infinite distress and difficulty to their parents, even when their actions are not criminal, and neither society nor the law makes any proper provision to meet the facts of their cases. A very sad case occurred in Scotland lately, where a boy of 16 in a great public school set fire on two occasions to the buildings from a motive utterly inadequate and ridiculous. He was made to play football, disliked it much, and one day had a kick on the head which stunned him somewhat, and seemed to cause a stupidity at lessons and a change in his mental condition. He had always been soft, and had associated with boys younger than himself, but in some respects had good mental aptitudes. He committed the act of arson under most peculiar circumstances, telling another boy he was going to do so. He was most active and cool in trying to put out the fire. He confessed his crime afterwards, but evidently had no proper realisation of its true nature nor of its possible awful consequence in causing the death of himself and of many of the other boys, and he had no realising sense at all of the legal position in which he was placed. When examined by Dr. Littlejohn and myself he could not be made by anything we could say, and we both tried hard to move him, to feel his true position or the distress he had caused his relatives. When brought up before the sheriff after the crime he was heedless as a child, and enjoyed the "holiday" immensely when out on bail before his trial. But he did not come within any definition of "insanity" at present recognised by the law, and therefore was punished as an ordinary criminal. I have known many lads and girls grow up past puberty who could not be made to realise the moral relation between the sexes, or even the ordinary decencies of life, any more than if they had been lower animals. Everybody knows the cases of inspired idiots who exhibited some wonderful special faculty, such as music, arithmetic, or painting. With very ordinary intellect Zerah Colburn, the American prodigy, could, at eight years of age, by working mentally, being quite ignorant of logarithms, raise a number of one figure to the twelfth or thirteenth power, and do other amazing arithmetical problems.

